# Polder maps: improving OMIT maps by excluding bulk solvent

**DOI:** 10.1107/S2059798316018210

**Published:** 2017-02-01

**Authors:** Dorothee Liebschner, Pavel V. Afonine, Nigel W. Moriarty, Billy K. Poon, Oleg V. Sobolev, Thomas C. Terwilliger, Paul D. Adams

**Affiliations:** a Molecular Biophysics and Integrated Bioimaging Division, Lawrence Berkeley National Laboratory (LBNL), Berkeley, CA 94720, USA; b Bioscience Division, Los Alamos National Laboratory, Los Alamos, NM 87545, USA; c Department of Bioengineering, University of California Berkeley, Berkeley, CA 94720, USA

**Keywords:** OMIT maps, polder maps, ligand validation, bulk solvent, weak density, residual (difference) Fourier synthesis, *PHENIX*

## Abstract

The crystallographic maps that are routinely used during the structure-solution workflow are almost always model-biased because model information is used for their calculation. As these maps are also used to validate the atomic models that result from model building and refinement, this constitutes an immediate problem: anything added to the model will manifest itself in the map and thus hinder the validation. OMIT maps are a common tool to verify the presence of atoms in the model. The simplest way to compute an OMIT map is to exclude the atoms in question from the structure, update the corresponding structure factors and compute a residual map. It is then expected that if these atoms are present in the crystal structure, the electron density for the omitted atoms will be seen as positive features in this map. This, however, is complicated by the flat bulk-solvent model which is almost universally used in modern crystallographic refinement programs. This model postulates constant electron density at any voxel of the unit-cell volume that is not occupied by the atomic model. Consequently, if the density arising from the omitted atoms is weak then the bulk-solvent model may obscure it further. A possible solution to this problem is to prevent bulk solvent from entering the selected OMIT regions, which may improve the interpretative power of residual maps. This approach is called a polder (OMIT) map. Polder OMIT maps can be particularly useful for displaying weak densities of ligands, solvent molecules, side chains, alternative conformations and residues both in terminal regions and in loops. The tools described in this manuscript have been implemented and are available in *PHENIX*.

## Abstract 2

Residual OMIT maps can be improved by the selective exclusion of bulk solvent from the OMIT region.

## 1. Introduction   

OMIT maps (Bhat & Cohen, 1984 ▸) are a widely used tool to verify whether a certain region of a model in a crystallographic map has sufficient density to justify its presence in the model. An OMIT map is calculated by excluding the atoms in question from the model and is especially useful to verify the presence of ligands, solvent molecules, alternative conformations and residues with weak electron density. Various kinds of OMIT maps have been proposed (Bhat, 1988 ▸; Hodel *et al.*, 1992 ▸; Vellieux & Dijkstra, 1997 ▸; Gunčar *et al.*, 2000 ▸; Terwilliger, Grosse-Kunstleve, Afonine, Moriarty, Adams *et al.*, 2008 ▸; Pražnikar *et al.*, 2009 ▸). These maps have their advantages and disadvantages. In the case of ligands and alternative conformations, it is desirable to first build and correct the rest of the model before placing a ligand or building difficult-to-interpret residues (‘discovery map’; Tronrud, 2008 ▸).

**Figure 1:**
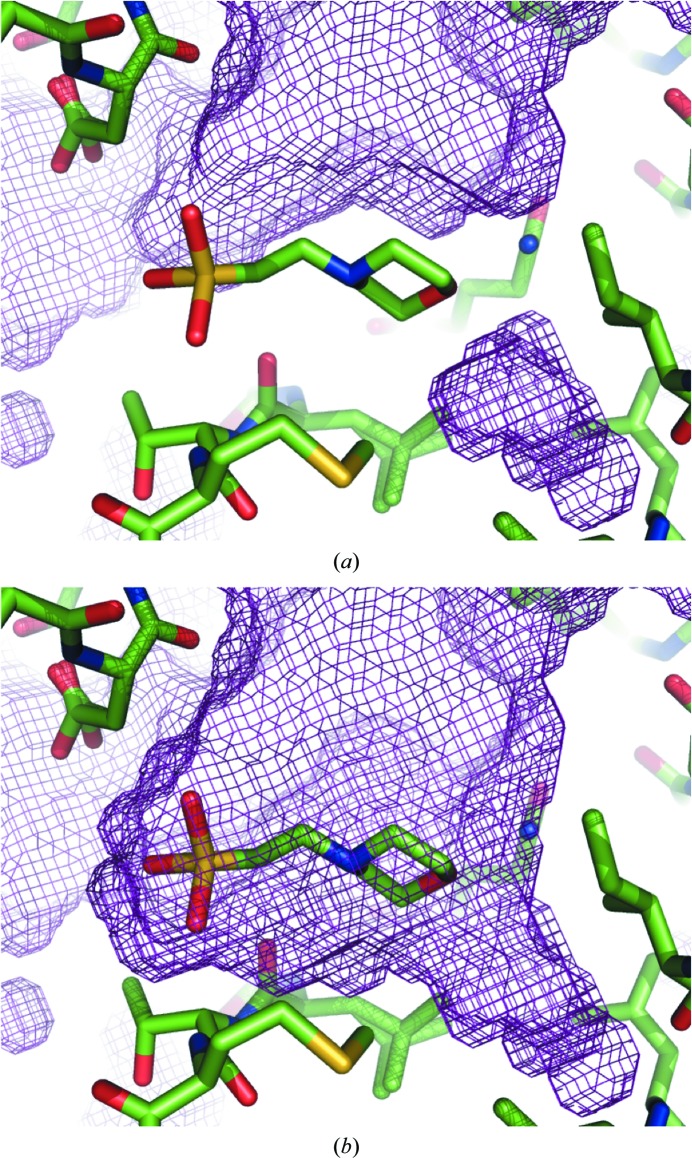
Illustration of how the bulk-solvent mask changes when a ligand (MES 88, PDB entry 1aba) is included (*a*) or excluded (*b*) in its construction. Exclusion of the ligand results in the bulk solvent filling the area previously occupied by the MES molecule.

The utility of OMIT maps may be limited by the flat bulk-solvent model that is commonly used in macromolecular crystallographic packages such as *CNS* (Brünger *et al.*, 1998 ▸), *REFMAC* (Murshudov *et al.*, 2011 ▸) and *PHENIX* (Adams *et al.*, 2010 ▸). This model assumes a constant solvent density (typically 0.2–0.6 e Å^−3^) anywhere in the unit-cell volume that is not occupied by the current atomic model (Jiang & Brünger, 1994 ▸). The areas of the unit cell that are not interpreted in terms of an atomic model are filled with the bulk solvent by constructing a binary mask set to 0 inside the atomic model and 1 elsewhere (Phillips, 1980 ▸; Fokine & Urzhumtsev, 2002 ▸; Afonine *et al.*, 2005 ▸; Weichenberger *et al.*, 2015 ▸). If the ligand is removed from the model, the region where it was modeled will be filled with bulk solvent, as shown in Figs. 1 ▸(*a*) and 1 ▸(*b*). This may diminish regions of weak density, complicating their interpretation.

**Figure 2:**
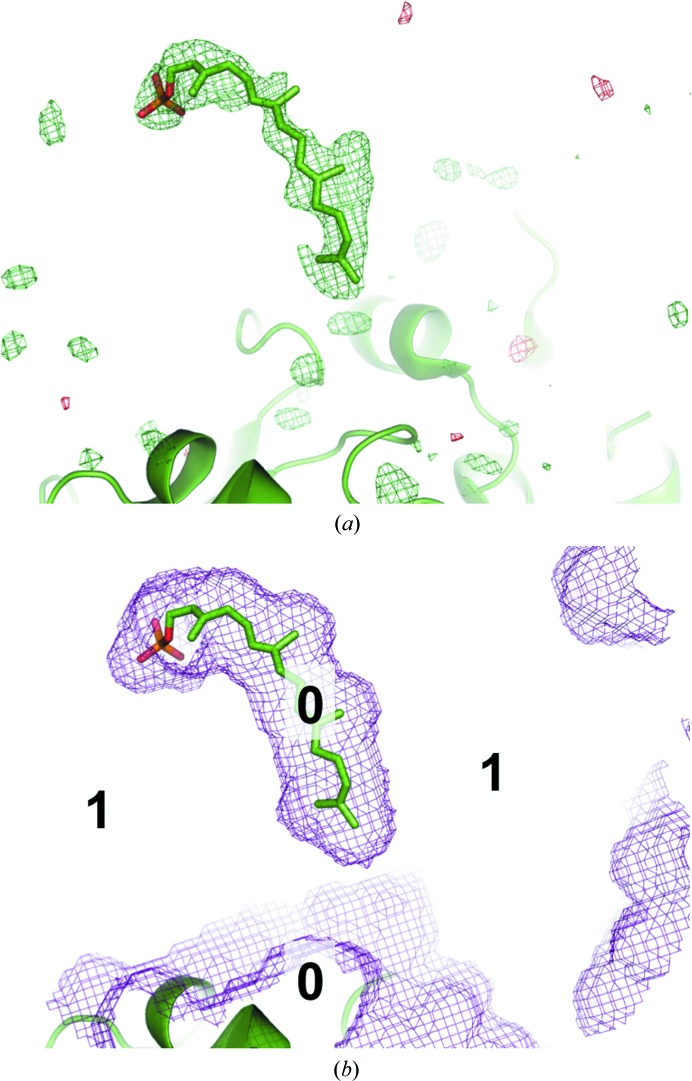
(*a*) A ligand molecule (GRG, PDB entry 4opi) is moved to an arbitrary location in the bulk-solvent region devoid of any electron-density peaks that could justify its position. The volume around the ligand is excluded from mask calculation and its contribution to the structure factor is ignored. The *mF*_obs_ − *DF*_model_ map contoured at 3σ shows strong positive density that follows the shape of the molecule. (*b*) Example of the mask when ligand is taken into account for mask computation but not for structure-factor calculation (biased map). The protein region is marked 0 and the bulk-solvent mask is marked 1. The mask employed in (*b*) was used to compute the difference map in (*a*).

Two typical ways of computing an OMIT map are worth mentioning. One is to remove the atoms in question from the model. In this case, the region containing atoms before their omission will be considered as part of the solvent region. Such a map would not be a true OMIT map since the flat bulk-solvent model will be placed into this region. This would obscure the signal from any structured atoms in this region, making it relative in comparison with the bulk-solvent density. Alternatively, one may restrict filling this region with the solvent model. Typically, this is performed by keeping the atoms in question in the model and setting their occupancies to zero so that they do not contribute to the scattering and also demarcate the solvent mask in this region as nonsolvent (see, for example, Fig. S1*b* in Choudhary *et al.*, 2014 ▸). The problem with this approach is that the residual map calculated using such a model is likely to show positive density mimicking the shape of the excluded region. This density may arise from the pure bulk solvent or structured atoms or a mixture of these, and owing to the way that this map is calculated it is not possible to discern the source of this density. To illustrate this, a ligand was placed into an arbitrary location in the bulk-solvent region, its occupancy was set to 0 and a residual *mF*_obs_ − *DF*_model_ map was calculated (Fig. 2 ▸ *a*). Rather strong positive density clearly follows the molecule. Obviously, this density reveals the bulk solvent (Fig. 2 ▸ *b*) and not the ligand. In the following, we refer to this kind of map as a biased residual OMIT map.

A possible solution to this problem is to realise that the model of the crystal content consists of two major contributions: atomic model and non-atomic model (bulk solvent). Therefore, both the atoms and the bulk solvent should be excluded from the OMIT map calculation and not just the atomic model. Several options have been proposed to deal with the bulk solvent in calculating OMIT maps, such as truncating the low-resolution data and not using the bulk-solvent model at all, or using alternative bulk-solvent models that do not employ *a priori* modeling (masking), such as the exponential scaling (Babinet) model (Moews & Kretsinger, 1975 ▸; Tronrud, 1997 ▸). Both options are problematic. Truncating the low-resolution data will degrade the quality of the map (Lunin, 1988 ▸; Urzhumtsev *et al.*, 1989 ▸; Urzhumtseva & Urzhumtsev, 2011 ▸; Cowtan, 1996 ▸), while the Babinet bulk-solvent model is only valid at resolutions below 8–10 Å (Podjarny & Urzhumtsev, 1997 ▸). Yet another alternative is to define the OMIT region as larger than the molecule that one wants to identify and then fill the region with a regular grid of small scatterers (electrons or fractions thereof) and refine occupancies, *B* factors and coordinates restrained to their initial positions (Urzhumtsev, 1997 ▸). The program *BUSTER* (Bricogne *et al.*, 2016 ▸) allows the exclusion of regions from bulk solvent by processing an additional file which describes the binding site (without resembling the putative ligand). Furthermore, statistical treatment of non-uniformity of bulk solvent or as yet unmodeled regions has been discussed (Blanc *et al.*, 2004 ▸; Perrakis *et al.*, 1999 ▸; Roversi *et al.*, 2000 ▸).

In this manuscript, we describe a new approach implemented in the *PHENIX* software suite. The tool is called *phenix.polder* and the corresponding maps are referred to as polder maps. The term ‘polder’ was chosen as an analogy to the Dutch term for lowland reclaimed from the sea: polder is land gained by keeping water from penetrating the area. A polder map helps to enhance weak features in electron-density maps by keeping bulk solvent out of the area. Better features are achieved because the polder OMIT density is essentially raised by a constant value equal to the bulk-solvent electron density, compared with an OMIT density where the OMIT region is filled with bulk solvent. In a polder map, the density in the OMIT region is therefore not biased by the bulk solvent.

## 2. Methods   

**Figure 3:**
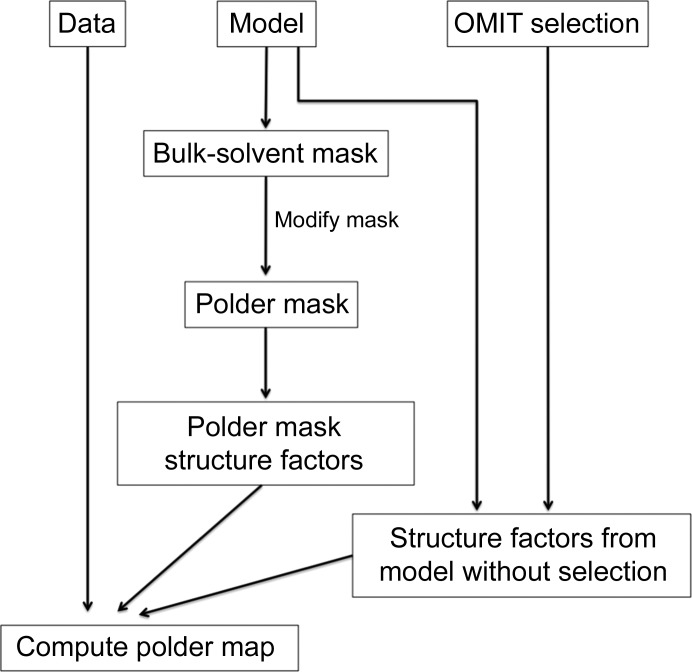
Working chart for *phenix.polder*. See text for details.

The calculation of polder OMIT maps consists of several stages (Fig. 3 ▸). Firstly, the OMIT region of the unit-cell volume is identified by selecting a group of atoms in the input model that are located in the OMIT region. An intersection of spheres of radius 5 Å around each selected atom is used to mark this region. The choice of 5 Å for the sphere radius is rather arbitrary and is based on two requirements. One is that the OMIT region needs to be large enough to avoid biasing the map by the shape of the masking atoms. The other is that the OMIT region should not remove too much scattering from the model because otherwise it will be damaging to the map. This is especially important for weak features in the map as they may be particularly susceptible to model deterioration. The results from calculations testing different radii for bulk-solvent mask exclusion are presented in the Supporting Information.

In the second step, a solvent mask is calculated from the atomic model that does not contain the selected atoms; this mask is then modified to exclude the solvent in the OMIT region defined above.

Finally, structure factors are calculated from the modified mask (‘polder mask’) and from the atomic model with the selected atoms omitted. These structure factors are then added together, scaled to *F*_obs_ as described in Afonine *et al.* (2013 ▸) and used for calculation of an *mF*_obs_ − *DF*_model_ map. *phenix.polder* produces a reflection file with two sets of Fourier map coefficients. One set corresponds to the polder OMIT map, and the other to the OMIT map where bulk solvent is allowed to penetrate the omitted region (the latter set of Fourier map coefficients is present for comparison). Biased residual OMIT maps were computed by including the OMIT atoms in calculating the bulk-solvent mask and calculating structure factors for the atomic model without the OMIT atoms. An example of the mask from this procedure (‘biased mask’) is shown in Fig. 2 ▸(*b*). To validate polder maps we have designed a numerical test, which is described in §[Sec sec5]5.

## 3. Results   

In this section, several examples of the utility of polder maps are presented.

### 3.1. Ligand density   

#### 3.1.1. Ligand GRG 502 in PDB entry 4opi   

**Figure 4:**
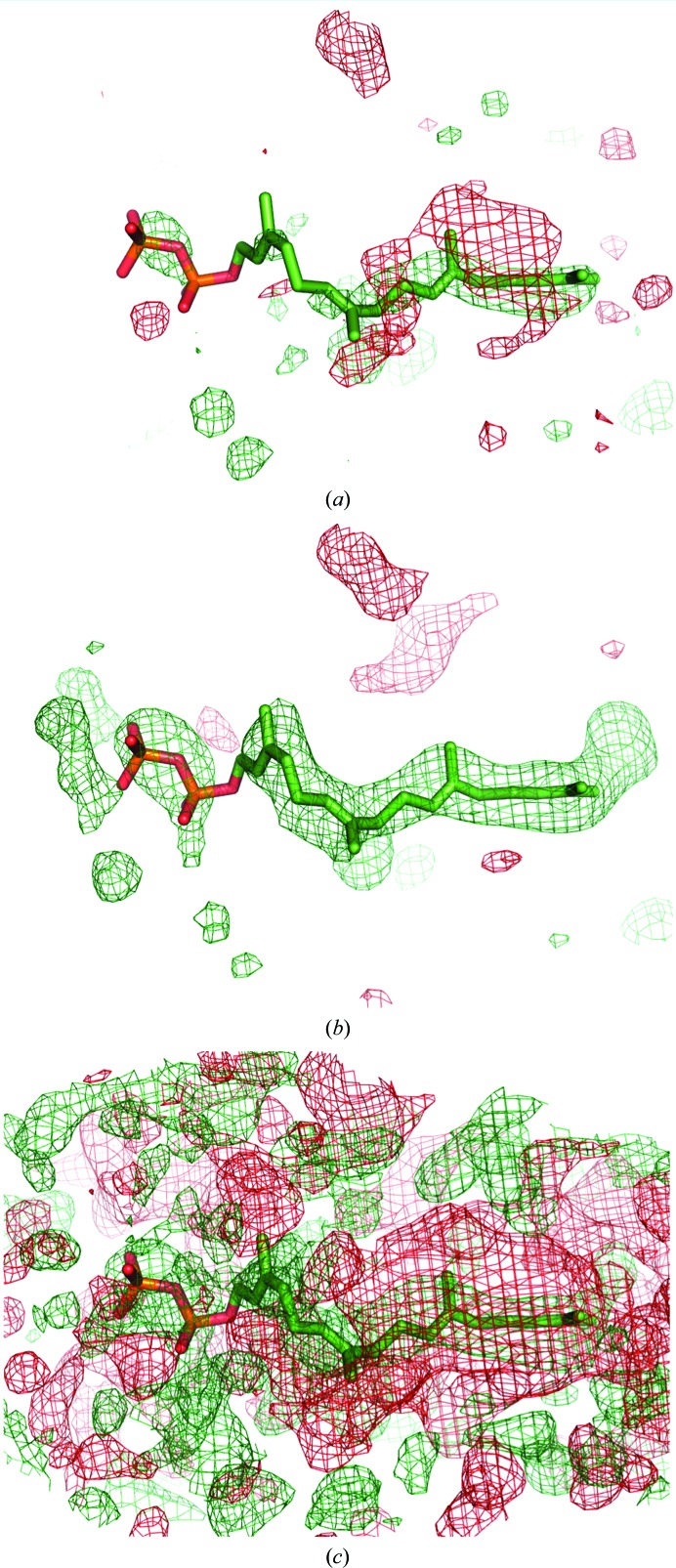
(*a*) OMIT map and (*b*) polder map for ligand GRG 502 in PDB entry 4opi. The positive and negative *mF*_obs_ − *DF*_model_ OMIT difference density contoured at 3σ is displayed in green and red, respectively. (*c*) OMIT map contoured at ±1.5σ, at which the ligand density has a similar shape to the polder map.

Figs. 4 ▸(*a*) and 4 ▸(*b*) show the OMIT map and the polder map for ligand GRG 502 from PDB entry 4opi (Kung *et al.*, 2014 ▸), respectively. In the OMIT map, only one phosphate group (left) and the tail located at the right side have positive density at a contour level of 3σ, whereas the center part of the molecule does not have density. At a similar contour level, the polder map shows density for the entire molecule (except for the O and C atoms next to the phosphate group). In order to obtain a similar shape of electron density as the polder map shows at a 3σ contour level, the contour of the OMIT map would have to be decreased to 1.5σ, which is much lower than what is usually accepted as a significant difference density peak (Fig. 4 ▸ *c*). The local correlation coefficient between the ligand model map and residual OMIT map (CC) is 0.70 and 0.75 for the OMIT map and the polder map, respectively, suggesting that the polder map is locally of better quality than the OMIT map. The minimum, maximum and mean values of the OMIT map and the polder map at the atomic centres of the ligand are summarized in Table 1 ▸. They are systematically larger in the polder map than in the OMIT map. For example, the average value is 4.14 e Å^−3^ in the former and 2.32 e Å^−3^ in the latter (reflection *F*000 was not accounted for here and everywhere else where absolute map values are reported). These higher map values are consistent with the results of visual comparison of the two maps, and with the expectation that a dominant effect of the polder map is to raise the level of the density by the bulk-solvent electron density in the OMIT region.

#### 3.1.2. Ligand MES 88 in PDB entry 1aba   

**Figure 5:**
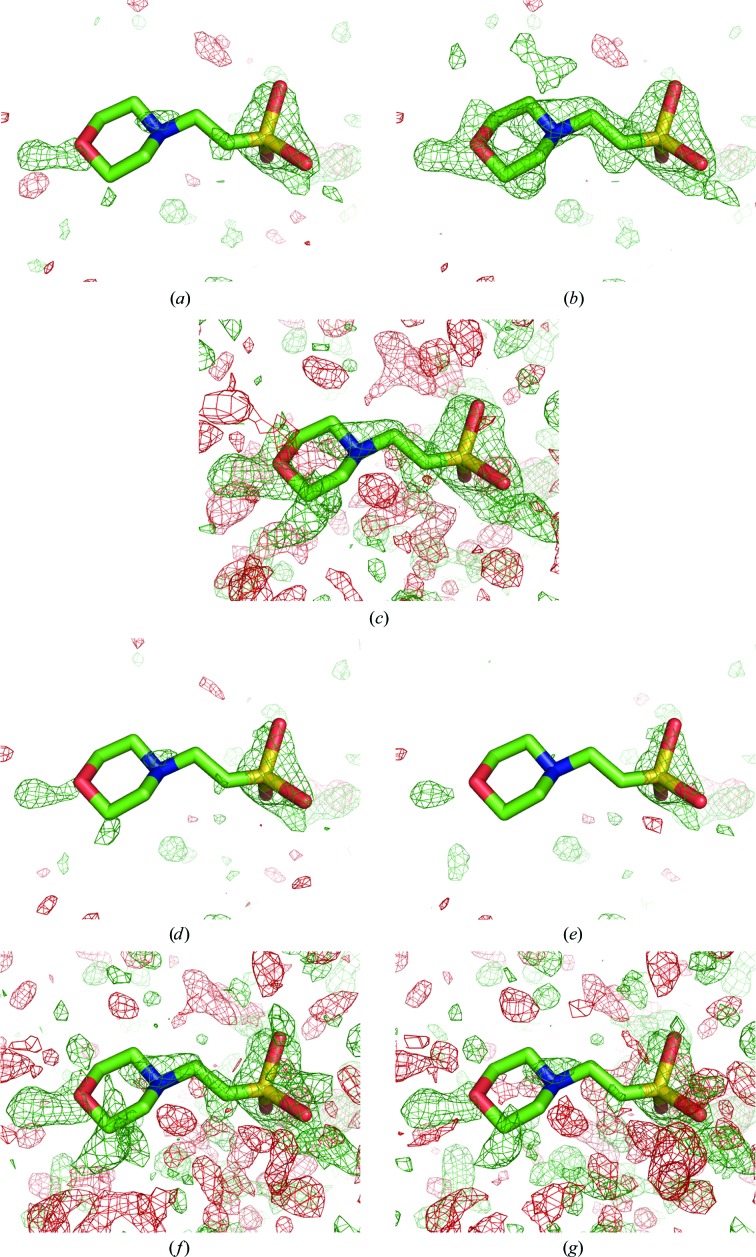
OMIT maps for ligand MES 88 in PDB entry 1aba. The positive and negative *mF*_obs_ − *DF*_model_ OMIT difference density is displayed in green and red, respectively. (*a*) OMIT map contoured at ±3σ. (*b*) Polder map contoured at ±3σ. (*c*) OMIT map contoured at ±2σ. (*d*) OMIT map using a Babinet solvent model (±3σ). (*e*) OMIT map not using any bulk-solvent model and truncating the data at 5 Å resolution (±3σ). (*f*) OMIT map using a Babinet model (±2σ). (*g*) OMIT map not using a solvent model and truncating at 5 Å resolution (±2σ).

Solvent molecules from the crystallization solution or the soaking or purification steps may be present in the crystal. As for ligands, strong evidence is needed to justify their presence in as yet un­modeled density. Figs. 5 ▸(*a*) and 5 ▸(*b*) show the OMIT map and the polder map for the solvent molecule MES 88 of PDB entry 1aba (Eklund *et al.*, 1992 ▸). In the OMIT map, there is only some density for the O, N and S atoms. Placement of the ligand appears difficult to justify looking at the electron density alone. In the polder map, there is difference electron density for the entire molecule; even the ring is correctly resolved. Additional positive electron-density peaks (on top of the ring) can be seen in the polder map. These peaks are likely to correspond to bulk solvent, which has been excluded around the MES molecule. In order to obtain a similar shape of difference density, the contour of the OMIT map has to be decreased to about 2σ (Fig. 5 ▸ *c*). The CC in the region of the MES molecule is 0.76 and 0.80 for the OMIT map and the polder map, respectively. The visual improvement in the density is therefore also supported by an increase in the local CC for the polder map. Furthermore, the map values are systematically larger for the polder map than for the OMIT map (Table 1 ▸).

#### 3.1.3. Ligand ABI 246 in PDB entry 1c2k   

**Figure 6:**
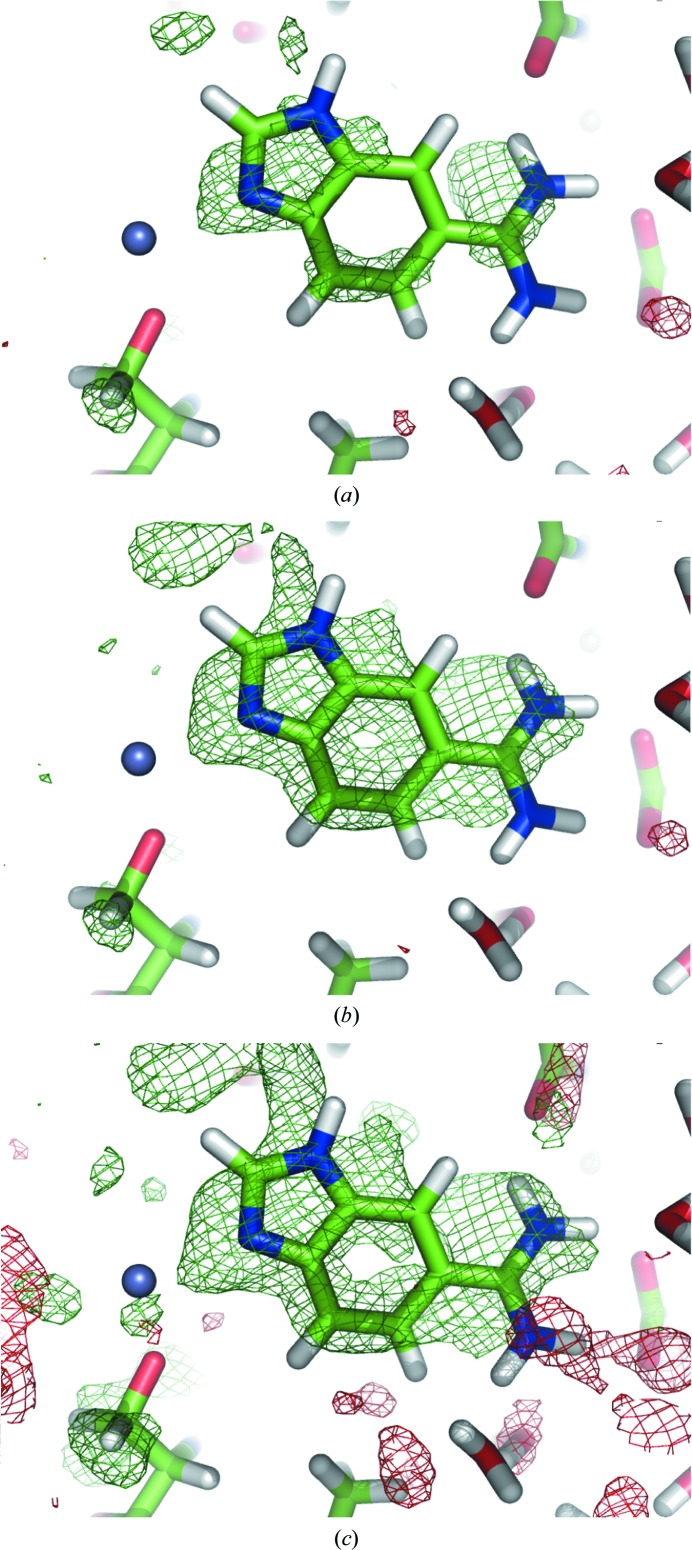
(*a*) OMIT map and (*b*) polder map for ligand ABI 246 in PDB entry 1c2k. The positive and negative *mF*_obs_ − *DF*_model_ OMIT difference density contoured at 3σ is displayed in green and red, respectively. (*c*) OMIT map contoured at ±2σ, at which the ligand density has a similar shape as the polder map. The gray sphere represents a Zn ion.

**Figure 7:**
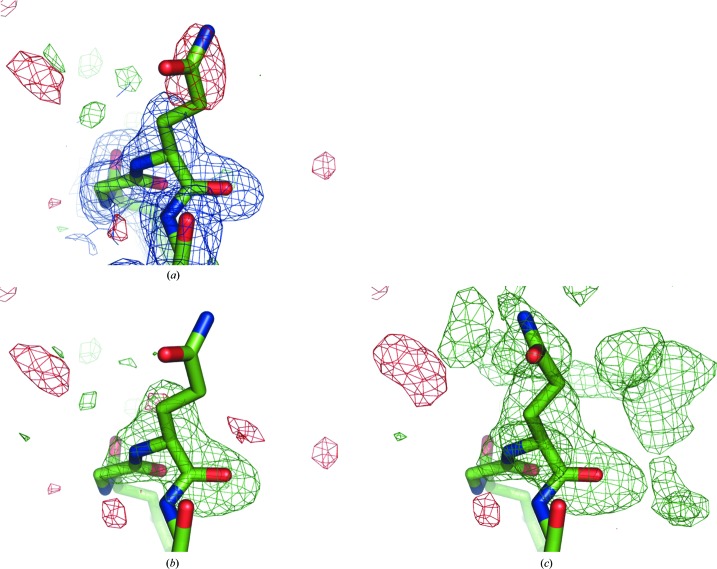
(*a*) Original 2*mF*_obs_ − *DF*_model_ (blue, 1σ contour) and *mF*_obs_ − *DF*_model_ maps, (*b*) OMIT map and (*c*) polder map for residue GlnH105 in structure 1f8t. The positive and negative *mF*_obs_ − *DF*_model_ difference density contoured at 3σ is displayed in green and red, respectively. In (*c*), the Gln side chain was real-space refined in *Coot*.

Figs. 6 ▸(*a*) and 6 ▸(*b*) show the OMIT map and the polder map of the ligand molecule 5-amidinobenzimidazole (ABI 246) from PDB entry 1c2k (Katz *et al.*, 1998 ▸). In the OMIT map, the density of the central six-membered ring is weak or missing, while the indole and the CN2 group are poorly resolved. In the polder map, the entire molecule is covered by density; the elongated shape of the residual peak around the CN2 group suggests that it slightly rotates around the C—C bond. In order to obtain a similar shape of difference density, the contour of the OMIT map had to be decreased to 2.1σ (Fig. 7 ▸ *c*). The map–model CC in the region of the ABI molecule is 0.64 and 0.69 for the OMIT map and the polder map, respectively. The mean map values at the atomic centers are 3.11 and 4.01 e Å^−3^ in the OMIT map and the polder map, respectively (Table 1 ▸), which is in agreement with the visual inspection, with more features visible in the polder map.

### 3.2. Side chains protruding into the solvent region   

The side-chain orientations of residues on the surface of the protein are often difficult to model. This is because they are highly mobile and are typically represented by an ensemble of conformations in crystal structures, and thus may have very weak or no density.

Fig. 7 ▸ shows the original 2*mF*_obs_ − *DF*_model_ and *mF*_obs_ − *DF*_model_ maps, the OMIT and the polder map for residue GlnH105 of PDB entry 1f8t (Fokine *et al.*, 2000 ▸). There is no density for the Gln side chain in the 2*mF*_obs_ − *DF*_model_ map, and the residual *mF*_obs_− *DF*_model_ map has a negative peak at the side chain position, suggesting that the modeled orientation is incorrect. In the OMIT map (Fig. 7 ▸ *b*) there is no density to indicate the orientation of the glutamine side chain either. In contrast, the polder map, while noisy, shows continuous V-shaped density suggesting a different orientation for the side chain. After real-space refinement in *Coot* (Emsley *et al.*, 2010 ▸), the side chain indeed fits well into the difference density (Fig. 7 ▸ *c*). The local CC improves from 0.77 in the OMIT map to 0.83 in the polder map. After real-space refinement based on the OMIT maps, these correlations increase to 0.80 and 0.87, respectively. This suggests that the new orientation describes the experimental data better. It should be noted that the map values at the atomic centers are systematically larger in the polder map.

## 4. Comparison of several methods to decrease the influence of bulk solvent in OMIT regions   

**Figure 8:**
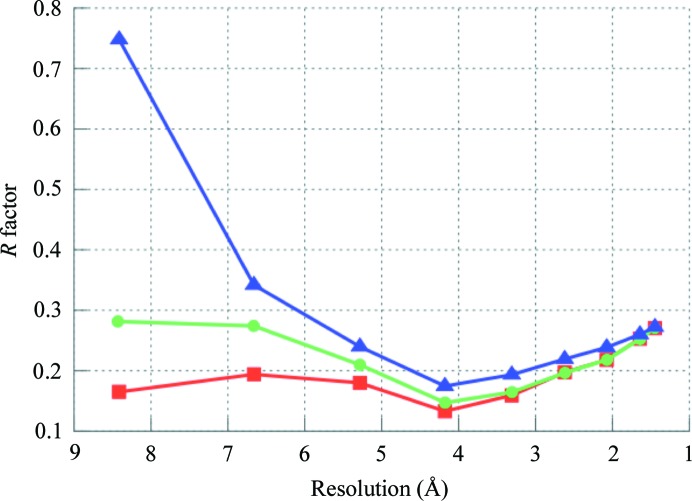
*R* factor *versus* resolution for PDB entry 1aba computed using the flat bulk-solvent model (red squares), Babinet solvent model (green circles) and no solvent model at all (blue triangles).

Several approaches have been proposed to decrease the influence of flat bulk solvent in OMIT maps: not using a bulk-solvent model at all and truncating the data at ∼5–6 Å resolution or employing a solvent model which does not use a mask, such as the Babinet model. Fig. 5 ▸ shows these maps for ligand MES 88 of PDB entry 1aba. For the map computed without using any bulk-solvent model, the resolution was truncated at 5 Å. This resolution cutoff was obtained by comparing the curves of the *R* factor *versus* resolution calculated using the flat bulk-solvent model and without using any bulk-solvent model (Fig. 8 ▸). The curves are similar up to 4 Å resolution and begin to diverge at about 5 Å.

Both maps, calculated using low-resolution truncated data and using the Babinet-based solvent model, show no improvement in the density for the MES molecule (Figs. 5 ▸ *d* and 5 ▸ *e*) at a contour level of 3σ. At a lower contour level (2σ; Figs. 5 ▸ *f* and 5 ▸ *g*) the map computed using the Babinet model (Fig. 5 ▸ *f*) is slightly superior to the truncated map (Fig. 5 ▸ *g*), but both are inferior to the OMIT map computed at a 2σ contour level (Fig. 5 ▸ *c*) and especially to the polder map (Fig. 5 ▸ *a*). Thus, the approach of truncating the low-resolution data to avoid bulk-solvent mask artifacts in residual maps is the least useful, which emphasizes the importance of low-resolution reflections for map quality. The Babinet model and the method ignoring low-resolution reflections are therefore not appropriate in cases of weak density and do not show an improvement compared with OMIT maps.

## 5. Validation of polder maps   

If the omitted region is surrounded by other atomic features of the model, such as a ligand in a compact binding pocket, the residual density revealed by the polder map might show behavior similar to biased OMIT maps: such density may correspond to either bulk solvent or ordered atoms. The reason is that in tightly packed environments bulk solvent or ligands are likely to mimic the shape of the pocket.

The susceptibility of the polder approach to bias owing to the shape of the ligand-binding pocket was tested by computing three polder maps. Two maps were computed using synthetic data (*F*_obs_ = |**F**_model_|), one assuming that the omitted atoms are present (m1) and the other assuming that the onitted atoms are not present (m2). The third map is a polder map using actual experimental data (m3). Local correlation coefficients (CC) and peak correlation coefficients (CC_peak_; Urzhumtsev *et al.*, 2014 ▸) were calculated between all three maps using map values from the OMIT region only. By the construction of the test, map m1 is expected to show omitted atoms and m2 is expected to show omitted bulk-solvent density. If the polder map m3 shows the omitted atoms it is expected to correlate best with map m1. If m3 shows the bulk solvent then it is expected to correlate best with map m2. However, if the omitted atoms are highly mobile (as manifested, for example, by large *B* factors) and/or the resolution is low, map m1 may be rather smeared and could resemble the bulk-solvent map, yielding high correlation with map m2. Also, Urzhumtsev *et al.* (2014 ▸) have demonstrated that CC_peak_ is more adequate for the comparison of three-dimensional functions. Considering pairwise comparisons of all three correlation coefficients, one can assess the confidence of interpreting the polder map in terms of an atomic model. Table 2 ▸ shows these correlation coefficients for all examples considered in this manuscript. Also, *phenix.polder* reports all of the abovementioned correlation coefficients.

### 5.1. Ligand LDT 320 in PDB entry 1us0   

As the examples in this manuscript consider weak OMIT densities, validation was also carried out for a ligand with very clear density in a high-resolution structure. The acetic acid molecule LDT 320 in PDB entry 1us0 (Howard *et al.*, 2004 ▸) is modeled with full occupancy (except for the Br atom, which has an occupancy of 0.94) and has very low disorder, with an average isotropic *B* factor of 4.2 Å^2^. For LDT 320, the pairs of correlation coefficients behave as expected. CC_m1m2_ and CC_m2m3_ are low (both have values of 0.24), while CC_m1m3_ is very high (0.99). The peak correlation coefficients follow the same trend, although the difference between CC_m1m2_/CC_m2m3_ and CC_m1m3_ is smaller (Table 2 ▸).

### 5.2. Ligand GRG 502 in PDB entry 4opi   

For the fictitious ligand placed in the bulk-solvent area in PDB entry 4opi, the correlation coefficient between m2 and m3 is the largest (CC_m2m3_ = 0.51), *i.e.* the map from calculated data without ligand correlates best with the experimental polder map (which, by the construction of this example, only contains bulk solvent). At the same time, the CC between m1 and m3 is very poor (CC_m1m3_ = 0.28), which also suggests that no ligand is present at this location.

For ligand GRG 502 in PDB entry 4opi, the CC between m1 and m3 is the largest (CC_m1m3_ = 0.77), while the other CCs are smaller (CC_m1m2_ = 0.66 and CC_m2m3_ = 0.65), thus suggesting that the binding cavity contains ligand and not bulk solvent.

### 5.3. Ligand MES 88 in PDB entry 1aba   

The CC between m1 and m3 for MES 88 is 0.80, while CC_m1m2_ (0.59) and CC_m2m3_ (0.48) are much lower. The CCs therefore strongly suggest that the ligand is present.

### 5.4. Ligand ABI 246 in PDB entry 1c2k   

**Figure 9:**
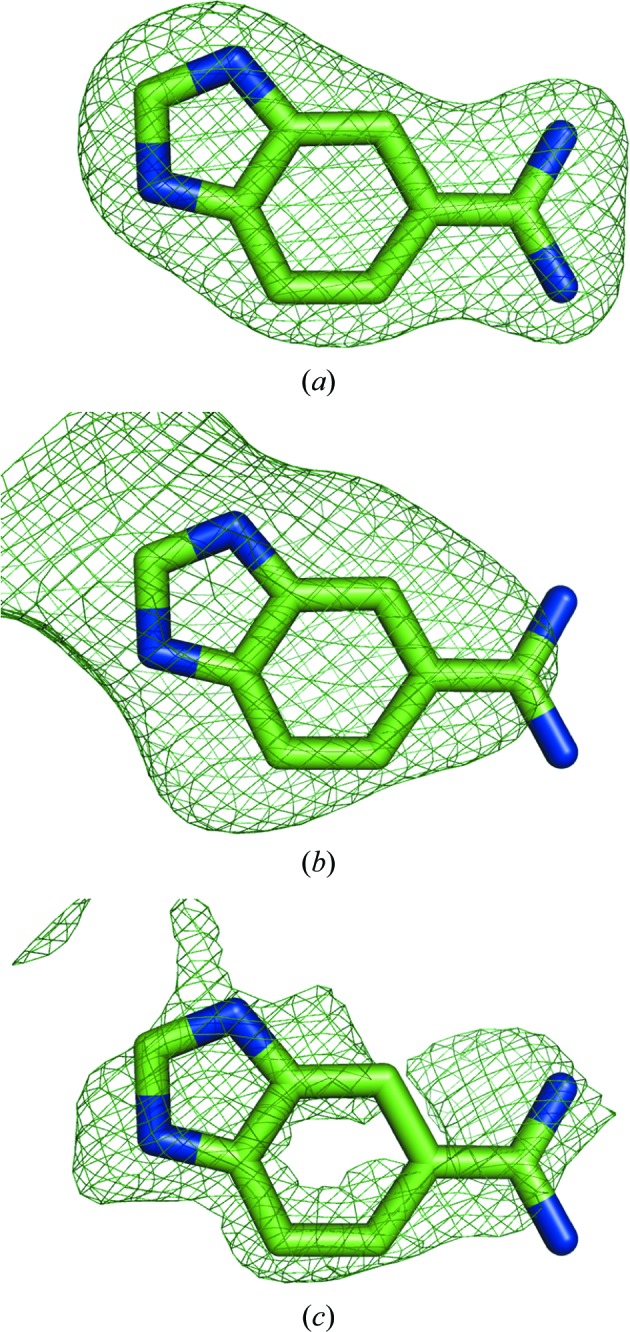
Trial polder maps of ligand molecule ABI 246 in PDB entry 1c2k used for the computation of correlation coefficients as described in §[Sec sec5]5: m1 (*a*), m2 (*b*) and m3 (*c*). All maps are contoured at 2.5σ.

For ligand ABI 246, CC_m1m3_ is larger (0.72) than CC_m1m2_ and CC_m2m3_ (0.63 and 0.64, respectively), thus favoring the ligand. However, the values of CC_peak_ are similar for CC_peak-m1m2_ (0.69) and CC_peak-m1m3_ (0.69) and only marginally differ from CC_peak-m2m3_ (0.63). Therefore, the electron density of this ligand should be interpreted with care. While numerical measures are not sufficient to decide whether the density belongs to ABI or bulk solvent, several considerations justify the placement of the ABI molecule. The polder electron density has the same shape as the ABI molecule; for example, it shows an ‘opening’ in the six-membered benzene ring of the indole group (at the 3σ contour level), and the two strongest peaks are at the N3 atom of the pyrrole ring and at the N1 atom of the CN2 group. Furthermore, the orientation of the molecule is such that it forms numerous hydrogen bonds to protein residues (for example N2—HH22⋯O_Asp189_), water molecules (for example N4—HN4⋯O_HOH260_) and a Zn ion (N3⋯ZN258). As the molecule displays rather strong disorder (*B*_iso_ of ∼50 Å^2^, occupancy 0.69), it is likely that its density resembles bulk solvent and therefore yielded high correlation to the bulk-solvent density in the binding region. Fig. 9 ▸ shows the three validation maps (m1, m2 and m3) for the ABI molecule. Map m1, which is based on calculated structure factors, clearly follows the shape of the molecule. Map m2, which represents bulk-solvent density, has a similar shape, although the density peak does not cover the CN2 group and also occupies the region further away from the indole group. This explains why m1 and m2 yield a relatively high correlation coefficient CC_peak_. The experimental map m3 shows greater resemblance to the calculated map m1 than to the bulk solvent in map m2, which is reflected by the CC_peak_ values, which are 0.69 and 0.63, respectively. It can be further noted that the ABI molecule was modeled with an occupancy of 0.69. Therefore, in 31% of the instances the binding pocket is filled with bulk solvent, which may explain the lack of a clear distinction between ligand and bulk solvent in this case.

### 5.5. Residue GlnH105 in PDB entry 1f8t   

The last example, residue GlnH105, shows a rather strong correlation between m1 and m3 (CC_m1m3_ = 0.88), whereas it is rather weak for the other maps (CC_m1m2_ = 0.17 and CC_m2m3_ = 0.09). The correlation coefficients between maps from synthetic data and from experimental data are therefore a good measure of the reliability of polder maps.

## 6. Comparison of simulated-annealing OMIT and polder maps   

**Figure 10:**
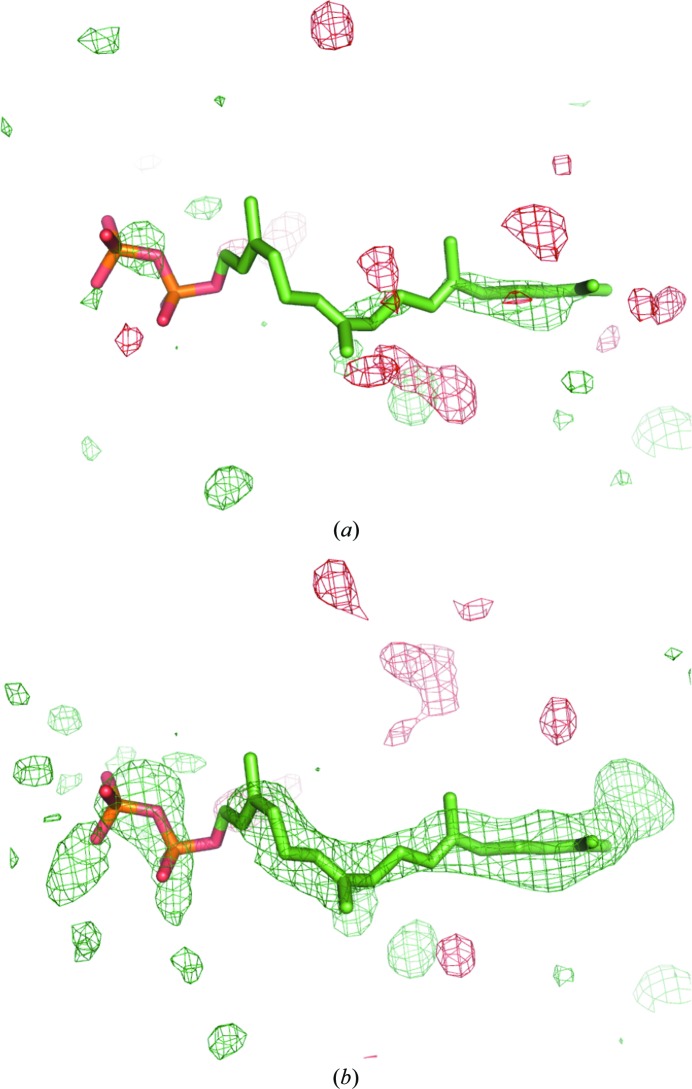
(*a*) Simulated-annealing OMIT map and (*b*) polder map for ligand GRG 502 in PDB entry 4opi. The positive and negative *mF*_obs_ − *DF*_model_ OMIT difference density contoured at 3σ is displayed in green and red, respectively.

It is often suspected that omitting atoms does not entirely remove model bias (Hodel *et al.*, 1992 ▸). It is therefore common to carry out several rounds of refinement, optionally adding simulated annealing (SA) to remove the ‘memory’ of the atoms to be omitted (Rupp, 2009 ▸; Terwilliger, Grosse-Kunstleve, Afonine, Moriarty, Zwart *et al.*, 2008 ▸; Brünger *et al.*, 1998 ▸). To compare the result of the polder procedure with a standard SA map, SA refinement was performed with the simulated_annealing=True option for the first macrocycle in *phenix.refine* (Afonine *et al.*, 2012 ▸) for model 4opi (without ligand GRG 502). The OMIT map and the polder map for ligand GRG 502 are displayed in Fig. 10 ▸. Similar to the results discussed in §[Sec sec3.1.1]3.1.1, the SA OMIT map (Fig. 10 ▸ *a*) has much less clear ligand density than the SA polder map (Fig. 10 ▸ *b*).

However, it is not recommended to carry out SA refinement routinely for polder maps. SA refinement may be appropriate for reducing model bias, but it has a limited scope of application. Firstly, during SA refinement the quality of the model may deteriorate if performed with regions of the model omitted. Generally, SA refinement is most appropriate at the initial stages of refinement (Adams *et al.*, 1999 ▸) as opposed to the final stages, when the polder map is likely to be needed. Since the aim of a polder map is to amplify weak features in electron-density maps, any potential worsening of the model is counterproductive. Finally, SA requires consideration of the refinement strategy, which is specific to the model, data and model-to-data fit qualities, and is not well suited to routine map calculation.

## 7. Conclusions   

The flat bulk-solvent model affects OMIT maps. To avoid its influence, a new tool, *phenix.polder*, has been developed as part of the *PHENIX* software suite. The tool calculates OMIT maps by not only excluding the selected atoms but also preventing the bulk-solvent mask from penetrating the region in question. As shown by several examples, *phenix.polder* is useful in cases where the density of the selected atoms is weak and possibly obscured by the bulk solvent. *phenix.polder* produces less biased maps than procedures in which the atoms are simply removed from the model or where the atom-selection occupancy is set to zero and included in the solvent-mask calculation. In the latter case, the resulting difference density can have a similar shape as the selected atoms. In the polder procedure, a larger volume from the bulk solvent is excluded and therefore prevents the misinterpretation of bulk-solvent density as OMIT density, making it a map-improvement technique that is suitable for parts of the structure with weak density. The program is available as from the command line as well as in the *PHENIX* GUI.

## Supplementary Material

Supporting Information.. DOI: 10.1107/S2059798316018210/ba5254sup1.pdf

## Figures and Tables

**Table 1:** Minimum, maximum and mean values of the electron density in e Å^−3^ at atomic centers in polder maps and OMIT maps

PDB code	Map	Minimum	Maximum	Mean
4opi	Polder	1.415	6.071	4.143
	OMIT	−0.262	4.544	2.321
1aba	Polder	1.847	13.440	4.591
	OMIT	0.252	12.734	3.454
1c2k	Polder	2.137	5.299	4.009
	OMIT	1.419	4.396	3.106
1f8t	Polder	3.200	12.564	7.425
	OMIT	1.504	11.737	6.219

**Table 2:** Local correlation coefficients (CC) and CC_peak_ between three polder maps: m1, m2 and m3 See §[Sec sec5]5 for details. Map 1 (m1), calculated *F*_obs_ assuming that the omitted atoms are present. Map 2 (m2), calculated *F*_obs_ assuming that the omitted atoms are not present. Map 3 (m3), polder map using experimental data.

		m1–m2		m1–m3		m2–m3	
PDB code		CC	CC_peak_	CC	CC_peak_	CC	CC_peak_
4opi	Fictitious ligand in solvent area	0.30	0.35	0.28	0.35	0.51	0.50
4opi	Ligand GRG 502	0.66	0.69	0.77	0.74	0.65	0.66
1aba	Ligand MES 88	0.59	0.69	0.80	0.72	0.48	0.54
1c2k	Ligand ABI 246	0.63	0.69	0.72	0.69	0.64	0.63
1f8t	Residue Gln105	0.17	0.30	0.88	0.77	0.09	0.16
1us0	Ligand LDT 320	0.24	0.53	0.99	0.85	0.24	0.47
