# Supplementary material for: Polder maps: improving OMIT maps by excluding bulk solvent
Source: Acta Crystallogr D Struct Biol. 2017 Feb 1;73(Pt 2):148–57. doi: 10.1107/S2059798316018210 (PMC5297918; doi:10.1107/S2059798316018210)
Supplement: Supplementary file 1 [file d-73-00148-sup1.pdf]

STRUCTURAL BIOLOGY

Volume 72 (2016)

Supporting information for article:

Polder maps: improving OMIT maps by excluding bulk solvent

Dorothee Liebschner, Pavel V. Afonine, Nigel W. Moriarty, Billy Poon, Oleg V. Sobolev, Thomas C. Terwilliger and Paul D. Adams

The radius within which the bulk-solvent is modified for polder maps (“polder radius”) should not be too large to prevent excluding too much of solvent scattering, thus worsening both the model-to-data fit and the map. On the other hand, the polder radius should not be too small to prevent shape bias from the excluded volume. However, finding the “optimal” radius for *phenix.polder* is not trivial. It could be defined in terms of “least biased” polder maps, or in terms of “best” validation CC values. The former is difficult to quantify. We attempt to address the latter by calculating polder maps and the concomitant validation correlation coefficients (local CC values) for several examples from the manuscript, varying the radius from 2.5 Å to 10 Å:

- 1ABA MES 88 molecule (realistic example)
- 4oPi GRG 502 molecule (realistic example)
- 1US0 molecule (clearly bound ligand)
- 4oPi fictitious ligand (no ligand at all)

We chose a minimum value of 2.5 Å because smaller radii will produce biased maps. The maximum radius tested is 10 Å, a larger radius is likely to deteriorate general map quality, especially if the OMIT region is located at the surface of the protein, where neighboring regions are likely filled with disordered bulk-solvent.

The CC values as a function of the radius are shown in Figs. S1-S4 for the four examples listed above. For the ligands in models 1ABA and 4oPi (Figs. S1 and S2), CC_{m1m2} and CC_{m2m3} are larger for radii between 2.5 Å and 3.5 Å, which is likely due to bias: for such small radii, the mask follows very closely the shape of the molecule. As the density of the ligands is weak, they have similar contour levels than the bulk-solvent, which leads to larger CC values. With increasing radius, CC_{m1m2} and CC_{m2m3} remain constant until about 9 Å (1ABA) and 7 Å (4oPi). For 4oPi, the CC values start fluctuating for larger radii, so the polder radius should in this case not exceed 7 Å.

For the clearly present ligand LDT 320 in model 1US0 (Fig. S3), the correlation coefficients are constant for different values of the radius. It can be noted that there is a slight increase for CC_{m1m2} and CC_{m2m3} for the radii 2.5 Å and 3.0 Å.

For the fictitious ligand placed in the bulk-solvent area in structure 4oPi (Fig. S4), the CC values fluctuate substantially for radii < 5 Å. For larger radii, CC_{m1m3} is systematically smaller than the other CC values, consistent with the fact that the ligand was placed into an arbitrary location in the bulk-solvent region. Therefore, a minimum radius of 5 Å should be used in this case.

From the above examples, it is concluded that a radius between 5 and 7 Å produces the optimum results. The radius is an optional input parameter in *phenix.polder* and can be changed by the user. The current code imposes a minimum value of 3 Å, to prevent the accidental calculation of “biased OMIT maps”.

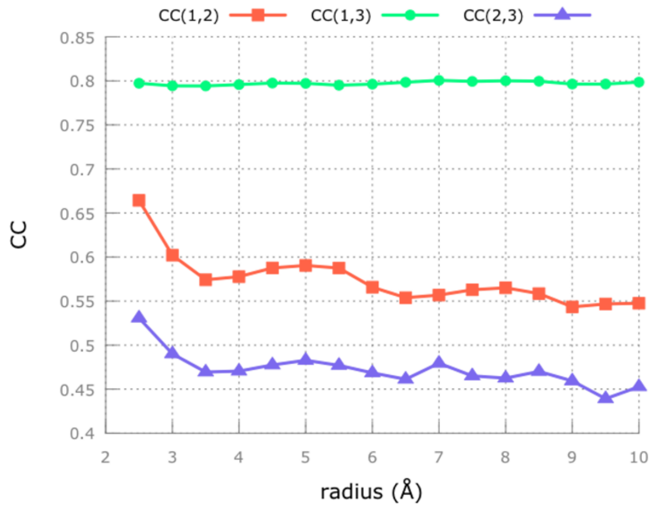

Figure S1 1aba polder map validation CC as a function of polder radius.

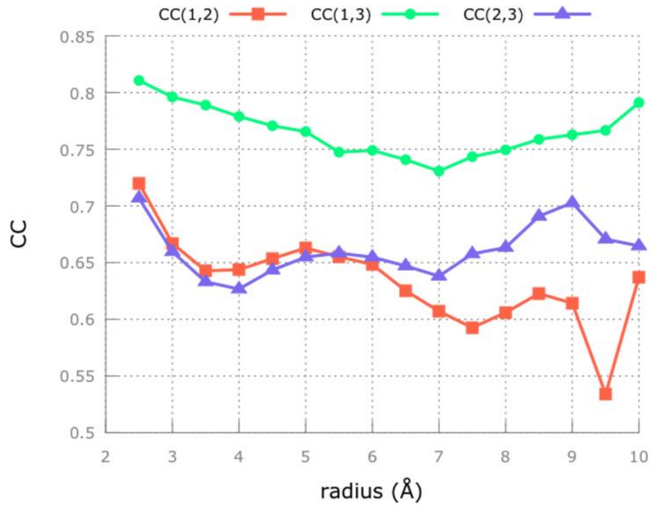

Figure S2 4opi polder map validation CC as a function of polder radius.

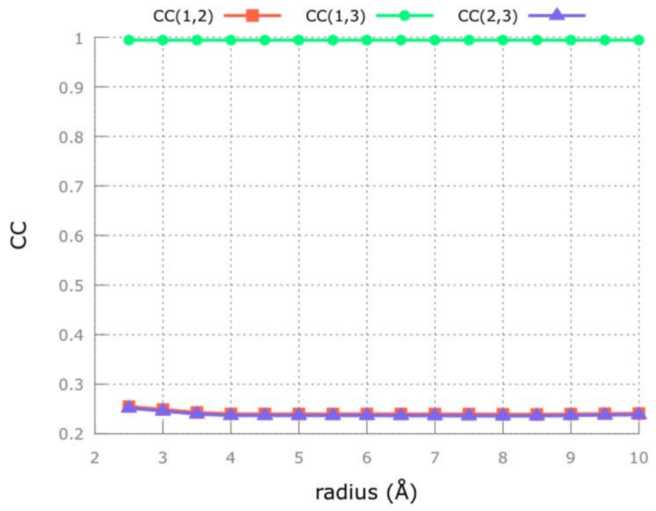

Figure S3 1us0 polder map validation CC as a function of polder radius.

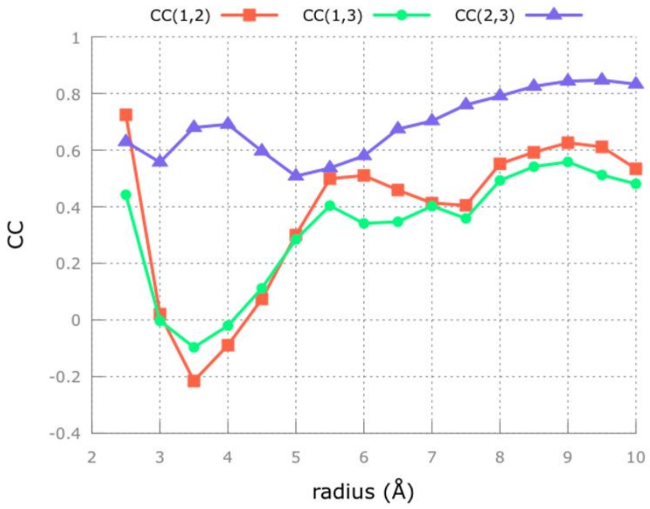

Figure S4 4opi polder map validation CC as a function of polder radius, using a ligand placed into the bulk solvent region.
